# Consumer Preference Heterogeneity Evaluation in Fruit and Vegetable Purchasing Decisions Using the Best–Worst Approach

**DOI:** 10.3390/foods8070266

**Published:** 2019-07-18

**Authors:** Stefano Massaglia, Danielle Borra, Cristiana Peano, Francesco Sottile, Valentina Maria Merlino

**Affiliations:** 1 Department of Agricultural, Forest and Food Sciences, University of Turin, Largo Paolo Braccini 2, 10095 Grugliasco, Italy; 2 Department of Architecture, University of Palermo, Viale delle Scienze 19, 90128 Palermo, Italy

**Keywords:** best–worst scaling, cluster analysis, consumer preferences, fruits and vegetables

## Abstract

This study assesses consumer preferences during fruit and vegetable (FV) sales, considering the sociodemographic variables of individuals together with their choice of point of purchase. A choice experiment was conducted in two metropolitan areas in Northwest Italy. A total of 1170 consumers were interviewed at different FV purchase points (mass retail chains and open-air markets) using a paper questionnaire. The relative importance assigned by consumers to 12 fruit and vegetable product attributes, including both intrinsic and extrinsic quality cues, was assessed by using the best–worst scaling (BWS) methodology. The BWS results showed that “origin”, “seasonality”, and “freshness” were the most preferred attributes that Italian consumers took into account for purchases, while no importance was given to “organic certification”, “variety”, or “brand”. Additionally, a latent class analysis was employed to divide the total sample into five different clusters of consumers, characterized by the same preferences related to FV attributes. Each group of individuals is described on the basis of sociodemographic variables and by the declared fruit and vegetable point of purchase. This research demonstrates that age, average annual income, and families with children are all discriminating factors that influence consumer preference and behavior, in addition to affecting which point of purchase the consumer prefers to acquire FV products from.

## 1. Introduction

The fresh fruit and vegetable (FV) market is an extremely widespread, complex, and global network. It is heterogeneous in terms of variety of products, with specific dynamics involving several stakeholders (producer, processor, and distributor) [1]. Together with the technological, biological, and industrial improvements that impact the FV sector, the exploration and introduction of new and different products has provoked a shift in this market, making it more globalized. The fruit and vegetable supply chain is characterized by important and dynamic logistics and management systems [2], which must respond effectively and promptly to changes that are both dictated by the market and often determined by consumer-led demands and needs. The decision-making role of consumers in defining end-product characteristics and supply-chain processes is evolving and has become one of the main drivers that needs to be analyzed in order to create a successful product. Given the heterogeneity and the multiplicity of the qualitative aspects that can characterize FV products, several studies have investigated consumption and consumer behavior in this market, including a multitude of choice attributes related to the experience and pleasure aspects of consumption [3]. One of the most debated aspects among the quality attributes of fruits and vegetables is human health. The consumption of FV has been linked to the prevention of several diseases [4]. It has been reported that people are currently consuming fewer FV products than the global recommended amounts, and therefore several food educational projects have been set up by means of national health promotion programs in schools and at many fruit and vegetable points of purchase [4]. In addition to human health-related aspects, several authors have analyzed the extrinsic, hedonic, and taste-related/sensory attributes that are important in the purchasing process [5,6,7,8,9]. For example, in [10], extrinsic quality attributes, such as brand, price, and packaging, were revealed to not affect the process of quality perception by consumers. On the contrary, sensory factors (i.e., intrinsic attributes of FV), such as the visual appearance, taste, freshness, color, aroma, texture, shape, nutritional quality, and crispness (for some products) were important attributes for fruit and vegetable quality evaluation [3,11,12]. Visual detection of fruit defects also plays a primary role in consumer attitudes and is correlated to the rejection of future purchases [13]. The guarantee of quality and safety standards of imported products, which is compliant with the European food safety legislations [14,15], has become a prerequisite for consumers. Thus, environmentally friendly quality parameters (low environmental impact, free from pesticides, socially oriented, and sustainable agriculture) now represent important quality attributes for consumers [16,17]. Attributes such as seasonality, locality, origin, and organic certification play a role in the selection of products from non-European countries where, in some cases, the use of pesticides, herbicides, and fungicides exceeds the safety thresholds established by the parameters of the European Union [18]. Other quality attributes such as freshness, seasonality, and price have been considered to be discriminating factors by consumers during FV purchases [19,20]. Freshness includes sensory properties (appearance, taste, smell, chemical, and physical) [21,22,23,24] and elements linked to the product origin (place of production, conservation technologies and processing, and packaging) [22]. Among the other FV product quality attributes, organic certification has proven to be a discriminatory consumer choice [25]. Finally, the product price is another important attribute for consumer decision making applied to all food products, including fruit and vegetables. In addition to the intrinsic/extrinsic product characteristics, research in the literature has also demonstrated that the consumer sociodemographic variables significantly discriminate their decision-making process [26,27], including the type of purchase outlet, which has proven to be related to an individual’s behavior and preference [28,29]. The aim of this paper was the exploration of consumer preferences and purchasing behaviors in Northern Italy, considering different fruit and vegetable sensory intrinsic/extrinsic attributes. The best–worst scaling methodology was applied to measure the relative importance of each attribute and a latent class cluster analysis was used for sample segmentation on the basis of consumer preferences. These methodologies have already been applied in several other studies in the agri-food sector [30,31] and enable the evaluation and comparison of the impact of the sociodemographic variables of the individual on the preferences and the selection of the fruit and vegetable point of purchase of the consumer.

## 2. Materials and Methods

### 2.1. Data Collection and Sample Characteristics

A choice experiment was conducted between 2017 and 2018 in two metropolitan areas of the Piedmont and Liguria regions in northwest Italy. Face-to-face interviews were conducted at six points of purchase (two mass retail chains and four open-air markets) in these geographical areas, alternating the day of the week (from Sunday to Saturday) and considering two time slots (from 9 a.m. to 1 p.m. and from 4 p.m. to 8 p.m.). During the intercept survey, individuals were randomly chosen, using the personal condition of “FV purchaser” as the only selection criterion. The first section included questions related to sociodemographic characteristics (gender, age, employment, education, and annual average income). The second section focused on fruit and vegetable purchasing and consumption behavior. In particular, this included examining the habitual points of purchase of fruit and vegetables (green grocer, mass retail channels, open-air market, food shop, organic food shop, purchasing groups, and producer). Consumer-declared preferences were collected in the third section, by means of the BWS methodological design implementation, which aimed to measure the relative importance placed by consumers on the previously selected 12 FV intrinsic and extrinsic attributes.

### 2.2. Best–Worst Scaling Design

The best–worst scaling (BWS) methodology is a method for collecting declared preferences which consists of requiring the respondent to select, among a series of attributes, the best and worst alternative from the list of proposals [32]. In applying this methodology, 12 extrinsic and intrinsic attributes (selection criteria) describing fruits and vegetables were selected from an in-depth literature research, which are presented in Table 1.

Using the Sawtooth Software (v.2.0.0.2, Orem, UT, USA), the 12 attributes were organized into four different versions of the questionnaire (integrated into the third section of the final questionnaire) and the order of the presented attributes varied in the different sets (nine per version). Each set contained four attributes and the single item appeared three times in the questionnaire, according to [70] and [39]. For each set of attributes, the respondent was required to indicate what was the most important (BEST) and what was the least important (WORST) item during their FV purchase. The experimental design followed the rule that, if the number of selected attributes is k and they are positioned in the subset C, then there are k(k–1)/2 pairs of BW (best-worst) and k(k–1)/2 pairs of WB (worst-best) associated with each subset [71]. Therefore, each choice set contains k(k–1) possible choice options (pairs BW and WB). In this way, the respondent chooses two maximum difference of preferences for each attribute set by providing more information than for single-choice options. This method is also called “maximum difference scaling”, because the chosen attributes should maximize the difference in utility made by a respondent on a preference scale. Finally, the BWS rating scales create an orderly ranking of items, explaining the level of relative importance appointed by each respondent for the single attribute using the average BW raw score (A-RS) or standard score [30,71,72]. This is calculated by dividing the BW score (best minus worst) [32] by the number of observations and the frequency that each attribute appeared in the four questionnaire versions. The A-RS is considered as the mean number of times each item was chosen as the most important or the least important by a single individual [73]. Among the main advantages, the BWS methodology allows measurement of the interview-declared preferences rather than, for example, the revealed preference which happens in traditional conjoint analysis. The BWS methodology allows for transformation of qualitative information into a real preference score (quantitative), avoiding the limitations, errors, and cognitive effort of the interviewed subjects, as in the case of, instead, being asked to classify (ranking) or declare a point (rating); for example, with the Likert scale [72,74,75].

The confidence limit applied in the estimation of the attribute scores was set at 95% and the standard deviation was used as a raw indicator of variability present within the sample. A two-tailed t-test was used to assess whether one attribute was preferred to another within the sample of respondents [76]. The rank of preferences derived from the best–worst scaling analysis and related to the expressed preferences of the whole sample allowed the application of latent class clustering (lClass) analysis, where the sample was divided into five clusters, according to the weight that the individuals assigned to the different attributes (average raw score values). The five selected groups of consumers were derived from the choices made between the four segmentations generated by the software. The most appropriate was reconciled by the one corresponding to the lowest BIC (Bayesian information criterion) value, in accordance with [42] (Table 2).

For cluster segmentation, the *p*-value for each attribute was calculated following a variance homogeneity test. The SPSS.21.0 software was used for the quantitative analysis.

## 3. Results and Discussions

### 3.1. Participant Description

A sample of 1170 respondents was collected for this study during the survey period: 684 were interviewed at large-scale retail stores and 486 were interviewed at open-air markets (Table 3).

### 3.2. Consumer Preferences for Fruit and Vegetable Intrinsic and Extrinsic Choice Attributes

The best–worst scaling analysis results supported the identification of the most important FV attributes considered by all interviewed consumers during purchase decision (Table 4).

Consumer choices during FV purchases were particularly influenced by the intrinsic factors of fruit and vegetable products: the origin having the highest average raw score (1.684), seasonality (average raw score 1.679), followed by the product freshness (average raw score 1.617). Other studies confirmed the importance given by consumers to the freshness attribute as a driver for purchasing fruit and vegetables [77,78]. Therefore, our study revealed that consumers, first and foremost, assess and pay attention to the product origin when buying FV, a quality attribute that focuses on the product identity and environmental and social sustainability, as well as the specific unique organoleptic characteristics [79,80,81]. Origin evaluation is closely linked to the product quality assessment, particularly since consumers generally consider a national product to be of better quality and safer than imported products [77]. The safety aspect also emerges as a discriminating factor linked to the origin of FV, characterized by a wide variety of products from different countries, including those outside the European Union. Often, in other similar studies, the analysis of the importance of local characteristics was carried out simultaneously with the seasonality attribute [63], together with sustainability, confirming the correlation between these quality attributes in FV products [59,82]. The price has also been shown to be an important attribute for consumers during FV purchases, evaluated as a quality indicator [13]. On the contrary, the worst values assigned by consumers for FV selection were attributed to extrinsic factors: organic certification (average raw score −1.438), variety (average raw score −1.077), and brand (average raw score −1.024). The negative evaluation of brand and special offers has also been confirmed by research in the existing literature [83,84]. However, our results regarding the non-important attributes for FV purchases were in contrast to other references in the literature [65,85].

### 3.3. Latent Class Analysis

The sample division of five clusters with different sizes is reported in Table 5. Each consumer group was named as a function of the most influential fruit and vegetable attributes considered during their purchase decision-making process (five groups). Only three FV quality attributes had positive values of average BW raw scores in all clusters (seasonality, freshness, and origin), while variety and brand were considered not important by consumers in all the five groups. The other seven attributes were differently considered among the different clusters.

#### 3.3.1. Preferences and Sociodemographic Variables of Different Consumer Clusters

##### Proposed Loyalty

**Figure 1:**
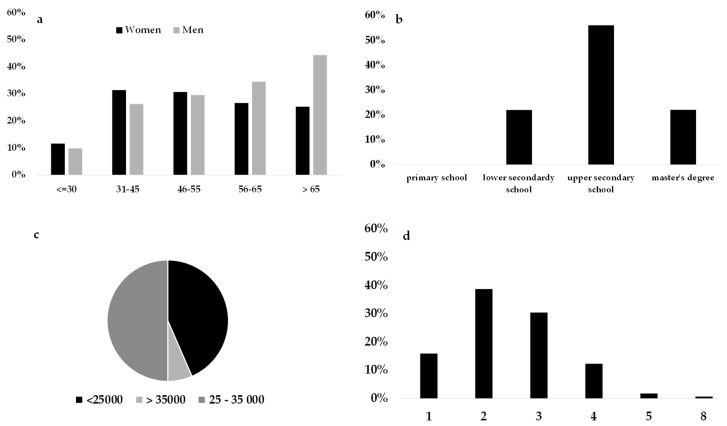
Sociodemographic characteristics of consumers belonging to the proposed loyalty cluster: (**a**) Gender and age group proportions, (**b**) educational level, (**c**) annual average income, and (**d**) number of family components.

Proposed loyalty cluster was the most highly represented group (25.5%). The sociodemographic variables of respondents belonging to this cluster are described in Figure 1. They were mainly women (67.6%) and were equally distributed among all age groups, with the exception of the youngest bracket. Meanwhile, men (32.37%) were well represented, mainly individuals 65 years and older. In accordance with the average age between individuals, this cluster mainly included employees and pensioners (the latter, presumably, men), and especially couples or families with a child. These respondents had a medium-high educational level, and a medium economic level. This cluster represents individuals who most likely entrust their selection of fruit and vegetables according to the market suggestions of what is in season, and who are attentive towards product freshness and origin. Variety, organic certification, local production, price, and special offers were not important at the time of purchase. These individuals expressed low importance towards the price and supply of the product, focusing their purchases on high-quality seasonal products. Moreover, the positive evaluation of the “certification” and “geographical indication labels” attributes, which occurred only in this cluster, further confirms that this attitude is oriented towards certified guarantees offered by the market.

##### Local Sensitive

**Figure 2:**
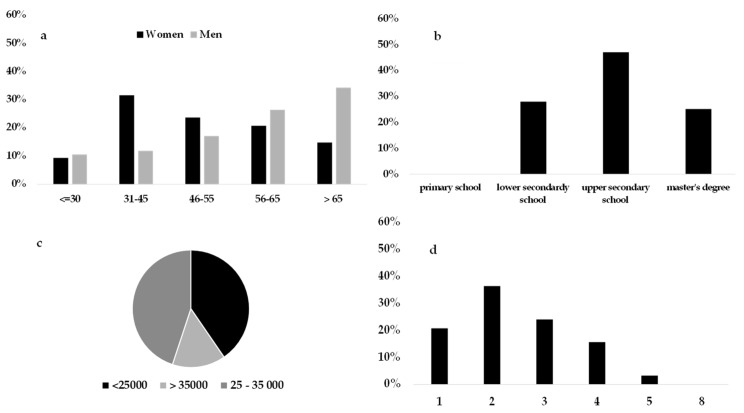
Sociodemographic characteristics of consumers belonging to the local sensitive cluster: (**a**) Gender and age groups proportion, (**b**) educational level, (**c**) yearly average income bracket, and (**d**) number of family components.

This cluster was the second largest group (25.2% of the respondents) and had a clear majority of women, as compared to men; however, in this case, the women were concentrated in the middle-young age group, while the men were more mature individuals. Again, average income was over 35,000 euros/year, with an educational level distributed between lower school and master’s degree (Figure 2). 

The local sensitive cluster had a majority percentage of three to four family members, but single individuals were also well-represented, in comparison to the other clusters. For these consumers, product price and promotion were not important during the FV purchasing process; however, seasonality linked to the proximity of production was fundamental in their choice. This was the only group where the consumers perceived the organic attribute positively, The interaction between “organic” and “locally grown” has also been studied and confirmed in [86], in addition to the preference of the local/organic combination by consumers [87], especially for young individuals. This was also confirmed in this cluster, since it was mostly represented by single women over 40 years old.

##### Price Sensitive

**Figure 3:**
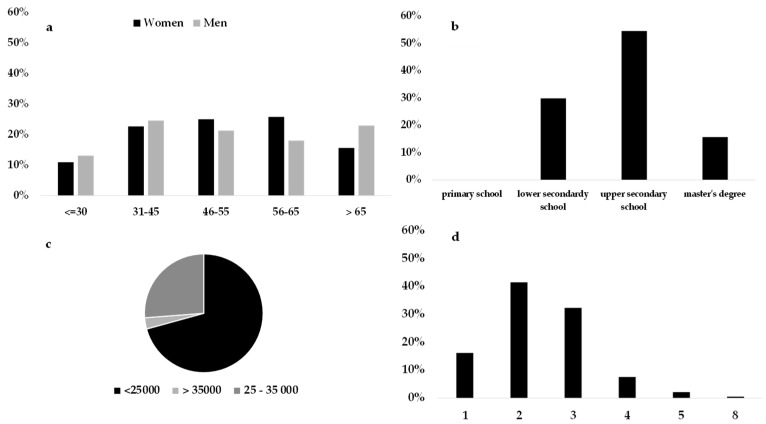
Sociodemographic characteristics of consumers belonging to price sensitive cluster: (**a**) Gender and age groups proportion, (**b**) educational level, (**c**) yearly average income bracket, and (**d**) number of family components.

The consumer profile of the price sensitive cluster represented 17.5% of the sample. Price sensitive consumers were the most evenly distributed between the two genders and age groups, emerging as the youngest cluster. Among the sociodemographic characteristics of these subjects, the average income emerged as the lowest out of the five clusters and the educational status was also at a low level (Figure 3). 

These individuals focused their attention on product price and special offers/promotions during the FV purchasing process. The average annual income was a discriminating factor for defining the behavior of these individuals, confirming the correlation between economic availability and willingness to buy [88,89]. The lack of economic independence or an inexperienced spending model (youngest consumer) could define the individuals within this cluster. They buy only in relation to their bank account, minimally considering quality attributes such as freshness or appearance. Among the quality attributes of the product, these individuals are attentive to seasonality (in season products cost less), confirming the propensity to choose a convenient product.

##### Undecided

**Figure 4:**
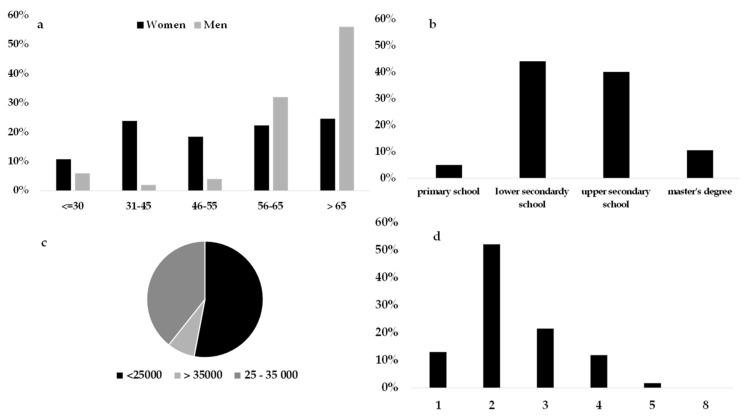
Sociodemographic characteristics of consumers belonging to the undecided cluster: (**a**) Gender and age groups proportion, (**b**) educational level, (**c**) yearly average income bracket, and (**d**) number of family components.

The undecided cluster (16.5% of the sample considered) was the cluster most represented by women (72%) who, most likely, did not devote much time to shopping, only purchasing generic FV products. The women were equally distributed among the different age groups, while the men were concentrated in the older age groups (Figure 4). The undecided individuals had a higher percentage of retirees among individuals (men) and of two-component families. Unlike other clusters, undecided individuals mainly represented two-component households with a low average annual income [89]. The preferences expressed by consumers were difficult to interpret in this group. In particular, these individuals were characterized by a widespread homogeneity of preference among the various factors of choice, buying what they found in the market at that particular time, looking at the origin (in some cases), but not exalting particular qualitative aspects of the product during their choice.

##### Value for Money

**Figure 5:**
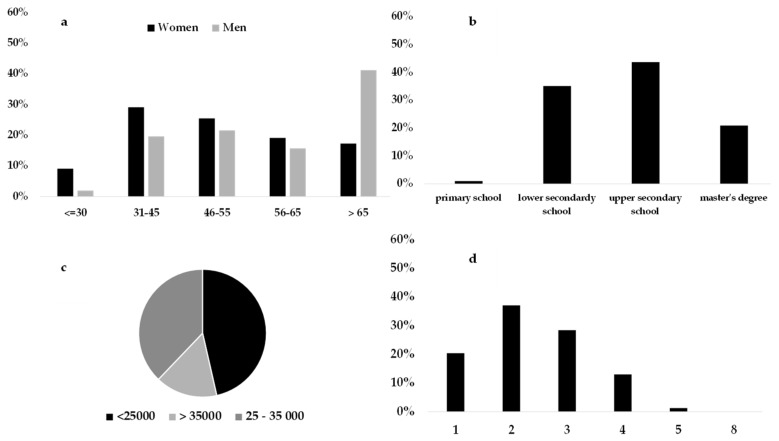
Sociodemographic characteristics of consumers belonging to value for money cluster: (**a**) Gender and age groups proportion, (**b**) educational level, (**c**) yearly average income bracket, and (**d**) number of family components.

The value for money cluster was the smallest group (15.2%). It consisted mainly of women in different age groups (from 30 to 65 years old), and mature men (over 65 years). This group had the highest percentage of individuals with high incomes, although, on average, we defined them at an average income level. In this group, there were 20% singles, and almost 40% of the individuals had one or two children in the household (Figure 5). 

These consumers expressed their preferences by interpreting the quality of the products provided by the experience, considering the freshness, appearance, and seasonality all as important, while not neglecting the price. The latter result is in line with the consumers in this cluster with the presence of children in households. In fact, it has been shown that special offers are often negatively evaluated in the fresh food products purchasing process, because it is perceived as a seller’s willingness to dispose of products with a lower quality standard [46,90].

#### 3.3.2. Fruit and Vegetable Point of Purchase Choice as a Function of Consumer-Declared Preferences

**Figure 6:**
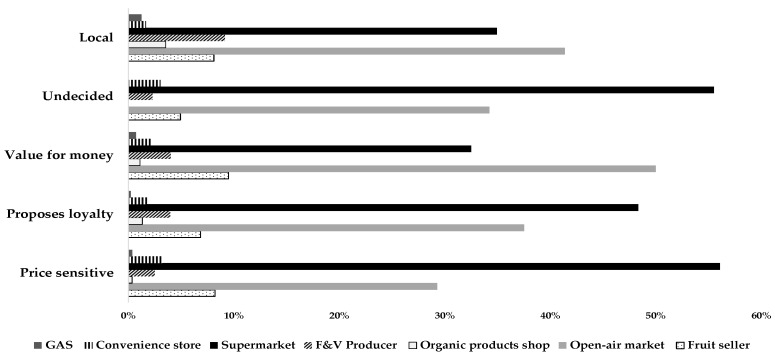
The points of purchase of fruit and vegetables chosen by consumers belonging to the five selected clusters.

Differences between consumer choices regarding the fruit and vegetable points of purchase emerged from habit exploration of the different consumer clusters (Figure 6).

Supermarkets were preferred by the price sensitive and undecided consumers. However, the sociodemographic factor that discriminated these two groups from the other clusters was the average low annual income. Therefore, it is possible to assume that the search for a product at a low price (price sensitive cluster) and the disinterested purchase (undecided cluster) can be satisfied by the offer from mass retail channels. The price sensitive group had the youngest respondents represented in the sample, confirming the attitudes of millennial consumers in preferring supermarkets for food purchases [91,92]. These consumers could coincide with a profile of buyers who are not attentive to locality or variety, in addition to brands and product certifications [48,83]. The choices of the undecided cluster coincided with the sociodemographic profile of active women, representing families composed of two members (husband and wife) who do not devote much time to shopping, but are also attentive towards the product origin, an attribute guaranteed by large and organized distribution. In the case of the proposed loyalty cluster, they bought FV mostly at the supermarkets, but also at open-air markets. The women from the proposed loyalty cluster represented families with children, highlighting their propensity to consider quality, pointing to extrinsic factors and seasonal products, relying on the proposals of the seller to protect the health of the family [93]. This also included the choice of supermarkets as a point of purchase for FV that guarantee information on the country of origin, the quality class, as well as higher prices which indicates greater guarantees of overall quality [94,95]. The research contained in [29] also pointed out that the need to satisfy consumer demand is reflected in an increase of shoppers at supermarkets. Therefore, characteristics such as appearance, sizing and packaging, and fast delivery, as well as permanent availability and a full range of products, are often requirements that cannot be met by local producers. A part of the proposed loyalty cluster is likely composed of more mature men who probably go to the open-air market or rely on the green grocer to buy products. In relation to the price sensitive and undecided clusters, a comparable sociodemographic composition is in the proposed loyalty cluster, which also has a medium to high average income as a discriminatory behavioral factor. Local sensitive consumers mainly bought from open-air markets and from producers, confirming that their propensity for local products is linked to a short production chain [3,96,97], but not excluding supermarkets from their choices. Open-air markets satisfy a need for familiarity, in addition to safety and sustainability (environmental, social, and economic) for local production [97,98]. However, the percentage of local sensitive consumers who bought from supermarkets was significant, because local origin guarantees can also be provided by mass retail chain standards. In addition, these individuals expressed a preference towards the organic attribute, confirming that organic shoppers are part of this consumer group [99]. The value for money group was distinguished by the higher percentage of individuals who bought fruit and vegetables at open-air markets and at green grocers. The presence of children in the family composition was also evident in this cluster. These consumers were comparable to the proposed loyalty cluster, in the search for a quality and seasonal product, but differed in the place of purchase [100,101]. For the value for money cluster individuals, the product price was also an important factor in the FV choice. Our results are confirmed by [95], which showed that the number of children and full-time work of the consumers are variables directly related to the evaluation of a higher food price perceived as an indicator of guaranteed quality.

## 4. Conclusions

This research explored the expressed preferences of a sample of consumers in northwest Italy towards extrinsic and intrinsic quality attributes of fruits and vegetables. In general, when choosing fruit and vegetables, consumers were mainly influenced by intrinsic and sensory product attributes such as origin, freshness, and seasonality.

The extrinsic aspects of fruits and vegetables were not deemed important for the choice of this type of product. However, the importance appointed to each attribute was heterogeneous among the different clusters of individuals identified in this study. The consumer cluster analysis considered not only individual preferences, but also their sociodemographic variables, allowing for an understanding of the connection between preferences and attitude formation, the intrinsic characteristics of individuals, and the purchasing behavior expressed at the choice of fruit and vegetable point of sale. Our results describe the fruit and vegetable consumer as being mainly women and middle-aged men, over 55 years of age. Through analysis of the clusters, it emerged that consumer choices are greatly influenced by average income and age, but, above all, the presence of children in the family leads the buyer to make more reasoned choices aimed at the search for quality, safety, and seasonality found in the local products of outdoor markets. This last result has underlined that the guarantee of the quality of fruit and vegetable products can also be ensured by producers, highlighting the importance of sustainability linked to local production systems and short supply chains, as well as guarantee of freshness (an intrinsic aspect of the product among the most important for the choice). Attributes such as local origin, seasonality, and freshness describe a safe product, even for the youngest members of the family. On the contrary, the economic resources of the families have proven to be discriminatory during the purchase of FV, directing low income consumers towards the purchase of generic products with no particular characteristics. Family consumers were also the most likely to buy fruit and vegetables in supermarkets. The guarantee of a better quality of the product was, therefore, perceived as being linked to a higher cost, which can be found in outdoor markets and directly from the producer.

The limitations of this research may include the selection of attributes, which could be extended to a larger number. Although, this technique has made it possible to discriminate between the most important (origin and seasonality) and least important (organic and variety) attributes, consumer evaluations (credence and beliefs) could be deepened in future studies. In addition, the small geographical area and large metropolitan areas considered for research sampling represent the limits of our research; further research could be extended to other geographical areas to assess potential differences between the Italian regions and to compare consumer preferences of individuals belonging to small and large residential areas. Increased educational campaigns for school-age consumers could ensure a higher level of awareness of the benefits of consuming fruit and vegetables, in order to increase consumption even among younger consumers.

## Figures and Tables

**Table 1:** The 12 attributes of fruits and vegetables used for the best–worst scaling analysis and associated references.

Fruit & Vegetable Quality Attributes	Attribute Description	References
Brand (or seller)	Brand allows the consumer to identify and discriminate a product. The evaluation of the brand associates “research”, in relation to the “experience” of the characteristics of a product, together with information about the manufacturer.	[33,34,35]
Organic label	Organic certification has been recognized, in various studies, as an attribute that positively influences consumer choices at the time of purchase.	[36,37,38]
Quality certifications	This attribute is often related to greater product safety and wholesomeness that, in FV, often results in the reduction of pesticide risk.	[39,40]
Origin	Product origin is an intrinsic cue, linked to the consumer information acquisition of product/producer identification in addition to product quality assessment. Consumers believe in a higher quality of domestic food, in comparison to foreign products.	[41,42,43]
Price	Several studies have given evidence that the selection of fresh fruit and vegetables is often not influenced by price. Thus, considering a broader product category in our study, fruit and vegetables, price becomes relevant.	[16,44,45]
Offer	The evaluation of special offers (promotional prices) for fruit and vegetables is an important tool for this category of products, often characterized by medium–high prices. This factor often depends on the place of purchase and seasonality.	[46,47,48]
Appearance	The outward appearance of FV is one of the attributes that highly influences decisions at the time of purchase.	[46,49,50]
Local	Local production has a lower perception of risk, which helps to increase loyalty to local producers and to guarantee the sustainability of local companies.	[3,51,52,53,54,55,56,57]
Geographical indication label	The consumer attitude towards certified products (e.g., Protected Designation of Origin (PDO) and Protected Geographical Indication (PGI)) has been studied in various researches, evaluating different fruit and vegetable products and confirming the recognition by consumers of a higher quality compared to a conventional or commercial product, with higher organoleptic and taste properties.	[58,59,60,61]
Seasonality	The consumption of seasonal fruit and vegetables is associated with consumer choice behavior oriented towards an ecological product; one that avoids excessive packaging (such as tin and plastic) and waste, and which is considered to have a higher organoleptic quality and freshness.	[62,63,64]
Variety	Greater attention to this attribute differs by various types of consumers, especially in identifying targets that are more attentive to the variety (cultivars).	[65,66,67]
Freshness	Freshness is a very important quality criterion for FV acceptability. Consumer assessment of freshness of fruit and vegetables occurs through the analysis of sensory and visual aspects of the product appearance during purchase, but also during/after consumption.	[20,22,68,69]

FV: fruit and vegetable.

**Table 2:** Latent class clustering (lClass) analysis results: Comparison between the BIC (Bayesian information criterion) for cluster segmentation choice.

Groups	Replication ^1^	BIC
2	3	46257.960
3	4	45162.476
4	2	44564.793
5	4	44171.250

^1^ Number of replications of data coupling performed by software, based on initial setting (replication set = 5).

**Table 3:** Sociodemographic characteristics of the interviewed sample population (*n* = 1170).

Sample (*n* = 1170)					
Gender	male	31%	Employment	housewife	6%
	female	69%		unemployed	6%
Age	≤30	9%		employed	42%
	31–45	24%		self-employed	9%
	46–55	22%		retired	34%
	56–65	22%		student	3%
	>65	23%	Annual average income (€/year)	<25,000	40%
Education	primary school	6%		25,000–35,000	33%
	lower secondary school	26%		>35,000	8%
	upper secondary school	49%		n.d.	18%
	master’s degree	19%			

**Table 4:** Best–worst scaling count report (number of BEST and number of WORST), BW average raw score (A-RS), and standard deviation for each considered attribute.

Rank	Attribute	Number of Best	Number of Worst	BW Average Raw Score ^1^	Standard Deviation
1	Origin (Italian/foreign)	1521	345	1.6840	1.673
2	Seasonality	1563	278	1.6790	1.394
3	Freshness	1489	305	1.6170	1.052
4	Local	842	559	0.4030	1.461
5	Price	902	685	0.3700	2.003
6	Offer	798	831	−0.0260	1.913
7	Appearance	704	914	−0.2960	1.520
8	Geographical indication labels	444	1145	−0.8820	1.575
9	Certification	458	1102	−1.0110	1.407
10	Brand (or seller)	400	1166	−1.0240	1.227
11	Variety	319	1133	−1.0770	1.027
12	Organic	406	1383	−1.4380	1.918

^1^ A negative BW (best-worst) average raw score value is due to the attribute not commonly chosen as the best factor.

**Table 5:** The average BW raw score for each fruit and vegetable attribute resulting from the five clusters of consumers: price sensitive, proposed loyalty, value for money, undecided consumer, and local sensitive.

	Price Sensitive	Proposed Loyalty	Value for Money	Undecided Consumer	Local Sensitive	*p*-Value ^1^
Cluster dimension	17.50%	25.50%	15.24%	16.50%	25.26%	
*Attributes*		*Average BW raw scores*				
Offer	50.21	−20.27	−8.80	20.99	−45.83	0.966
Geographical indication labels	−30.48	18.74	−41.19	−32.70	5.71	0.548
Seasonality	11.91	45.11	51.82	23.17	54.17	**0.011**
Appearance	3.22	−11.70	39.14	−10.54	−39.42	0.777
Origin (Italian/foreign)	7.92	20.44	31.11	48.21	50.45	**0.017**
Price	64.25	−16.92	3.75	24.52	−36.37	0.675
Organic	−35.75	−39.25	−37.70	−51.79	6.03	**0.032**
Certification	−26.40	10.66	−33.82	−37.71	−6.84	0.107
Variety	−14.47	−38.02	−7.86	−30.22	−30.50	**0.012**
Local	−13.97	−28.89	−11.80	24.61	37.54	0.911
Brand (or seller)	−26.20	−0.65	−42.33	−6.48	−32.80	0.051
Freshness	9.76	60.75	57.67	27.93	37.87	**0.014**

^1^ The significant scores are highlighted in bold (*p*-value < 0.05).
